# A235 ILEAL MICROBIOME ALPHA DIVERSITY REMAINS LOWER IN ENDOSCOPICALLY AND HISTOLOGICALLY INACTIVE CROHN’S DISEASE COMPARED TO ULCERATIVE COLITIS OR HEALTHY CONTROLS

**DOI:** 10.1093/jcag/gwab049.234

**Published:** 2022-02-21

**Authors:** P A Olivera Sendra, K Borowski, S Nayeri, H Martinez-Lozano, H Leibovitzh, S Lee, C Hernandez-Rocha, M S Silverberg

**Affiliations:** Zane Cohen Centre for Digestive Diseases, Lunenfeld-Tanenbaum Research Institute, Sinai Health System, Toronto, ON, Canada

## Abstract

**Background:**

Crohn’s disease (CD) has been associated with a lower alpha diversity when compared to ulcerative colitis (UC) patients and healthy controls (HC), which also depends on disease location and endoscopic activity. However, it is unclear whether the resolution of histologic inflammation may influence mucosal alpha diversity in the ileum.

**Aims:**

To characterize the ileal mucosa-associated microbiome diversity in subjects with ileal predominant CD (iCD) in endoscopic (ER) and endo-histologic (EHR) remission, compared to subjects with colonic predominant CD (cCD), UC and HC, respectively.

**Methods:**

Data from a large cohort of subjects recruited at Mount Sinai Hospital Toronto (2009–2016) was analyzed. ER was defined as a segmental Simple Endoscopic Score for CD of <3 in the ileum. EHR was defined as ER with the absence of active histologic inflammation in ileal biopsies. CD patients were divided according to the Montreal classification into iCD (L1 and L3) and cCD (L2) and compared against UC patients without backwash ileitis and HC. Patients with history of ileocecal resection and/or antibiotic use at baseline were excluded. Microbial 16S rRNA gene was sequenced using the Illumina MiSeq and processed using QIIME2. Alpha diversity was measured using the Shannon index and compared using Kruskal-Wallis test and further pairwise Wilcoxon with Holm correction. An adjusted p-value < 0.05 was considered significant.

**Results:**

We included 35 CD patients with ileal ER, 81 UC patients and 32 HC. Among CD patients, 20/35 (57.1%) and 15/35 (42.9%) had iCD and cCD, respectively. Ileal mucosal alpha diversity was significantly lower in iCD patients in ER compared with that of UC patients (q=0.004) and HC (q=0.001). No differences in ileal mucosal alpha-diversity were seen between iCD than cCD patients (q=0.12). When histology was included to classify CD and UC patients as EHR, the ileal alpha diversity of iCD patients remained reduced compared to UC patients and HC (q=0.008 and q=0.002, respectively). Again, ileal mucosal alpha-diversity was not significantly lower in iCD than cCD patients among those in ileal EHR (q=0.24).

**Conclusions:**

Ileal mucosa of CD patients in ER and EHR have lower alpha diversity than UC and HC. These findings suggest that the ileal mucosa of CD patients remains dysbiotic despite achieving endoscopic and histologic remission.

**Funding Agencies:**

IBD Genetics Consortium

